# Bioactivities and industrial standardization status of *Ganoderma lucidum*: A comprehensive review

**DOI:** 10.1016/j.heliyon.2024.e36987

**Published:** 2024-08-29

**Authors:** Peng Wu, Chengyun Zhang, Yueyue Yin, Xiaobin Zhang, Qi Li, Lijingyi Yuan, Yahe Sun, Shuhua Zhou, Shanting Ying, Jiayan Wu

**Affiliations:** a BRICS Standardization (Zhejiang) Research Center, Zhejiang Institute of Quality Sciences, Hangzhou, China; b National Market Regulation Digital Research and Application Technology Innovation Center, Zhejiang Standardization Think Tank, Hangzhou, China; c Wencheng County Food and Drug Comprehensive Testing Center, Wenzhou, China; d Lishui Institute for Quality Inspection and Testing, Lishui, China; e TÜV NORD (Hangzhou) Co., Ltd., Hangzhou, China; f Anhui Guotai Zhongxin Testing Technology Co., Ltd., Hefei, China; g School of Chinese Medicine, Hong Kong Baptist University, Hong Kong SAR, China

**Keywords:** GL, Bioactivity, Human health, Industrial standardization

## Abstract

*Ganoderma lucidum* (GL) is a potent source of bioactive compounds with diverse nutritional and pharmacological benefits. Its popularity as a dietary supplement, herbal remedy, and wellness product is steadily on the rise. Furthermore, the standardized advancement of the GL industry has facilitated reliable sourcing of raw materials and quality control measures, enhancing its utilization and endorsement in the realms of nutritional science and pharmaceutical research. This article provides a comprehensive overview of the recent advancements in research pertaining to the bioactive components of GL, particularly polysaccharides (GLP) and triterpenes (GLTs) as well as highlights the latest findings regarding their beneficial effects on human diseases, including anticancer, antidiabetes, liver protection and other aspects (such as regulating gut microbiota, antioxidant, antimicrobial, antiinflammatory and immune regulation). Furthermore, we summarized the potential applications of GL in the food and pharmaceutical sectors, while also examining the current status of standardization throughout the entire industrial chain of GL, both domestically and internationally. These information offer an insight and guidance for the prospects of industrial development and the innovative advancement of GL within the global health industry.

## 1 Introduction

GL, a mushroom from the Ganodermataceae family and Ganoderma genus of fungi, has a history of several thousand years of traditional use for its health-promoting and longevity-enhancing properties [1,2]. Numeral researches have revealed that GL encompasses distinctive bioactive constituents, including polysaccharides (GLP) and triterpenes (GLTs), which contribute to its diverse pharmacological effects. These effects encompass but are not limited to antitumor, anti-inflammatory, hypoglycemic, lipid-lowering, immune-regulatory, neuroprotective, liver-protective, kidney-protective, and antiviral properties. As a result, GL plays a pivotal role in the management and prevention of a wide range of diseases [[3], [4], [5]]. GL exhibits a favorable safety profile with low incidence of side effects [6], in addition to being an effective medicinal plant, its unique potential nutritional characteristics have also led to its widespread application in the nutritional and health products and cosmetics industry [[7], [8], [9]].

The industrial cultivation of GL is globally widespread, with Asia serving as the primary production hub. There are approximately 200 worldwide varieties of GL, classified into six main color-based types: bright, black, green, white, yellow, and purple, each exhibiting varying functional activities. Furthermore, the standardized development of the GL industry has provided effective raw material supply and quality assurance for its application and promotion in food nutrition and pharmaceutical science, which also brings hope and exciting prospects for the innovative development of GL [10].

**Fig. 1:**
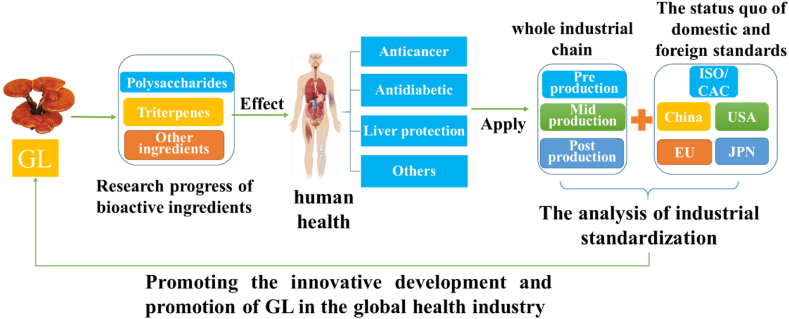
Research roadmap of this article.

Currently, there is a great deal of updated research on the extraction and analysis of key bioactive components of GL, such as GLP and GLTs, and their effects on human health, while the standardisation development of the industry is the key quality control assurance for GL to exert its pharmacological effects. Studying the key bioactive components of GL and exploring the association between them and the development of industrial standardisation is a novel perspective. This article provides a comprehensive overview of the recent advancements in research pertaining to the bioactive components of GL, particularly GLP and GLTs as well as highlights the latest findings regarding their beneficial effects on human diseases like cancer, diabetic, hepatic disease, and others (regulation of gut microbiota, antioxidant, antimicrobial, antiinflammatory, and immune regulation). Besides, we summarized the potential applications of GL in the food and pharmaceutical sectors, while also examining the current status of standardization throughout the entire industrial chain of GL, both domestically and internationally, and analyzes the pain points and future directions of its industrialization development. These results offer an insight and guidance for the prospects of industrial development and the innovative advancement of GL within the global health industry. The research roadmap of this article is shown in Fig. 1.

## 2 Research progress of GL

### 2.1 Bioactive ingredients of GL

**Fig. 2:**
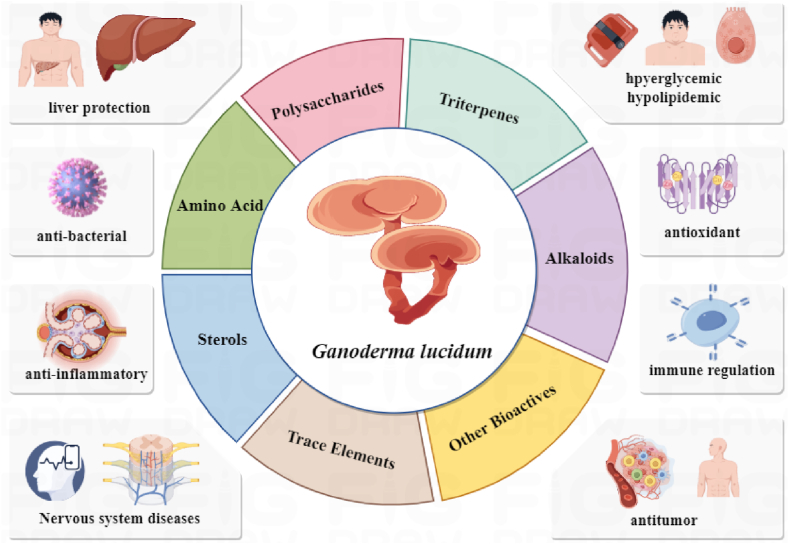
Bioactivities of the main active components of GL [25,26].

GL has a rich chemical composition, with non-volatile components that include fiber (59 %–65 %), carbohydrates (21.83 %–27.78 %), protein (7 %–8 %), fat (1.1 %–8.3 %), ash (0.72 %–1.77 %), and other constituents [11]. Table 1 summarizes and sorts out the composition of GL from several representative production areas. So far, the scientific community has isolated more than 400 bioactive compounds from GL fruiting bodies, hyphae, and spores, mainly including polysaccharides, triterpenes, steroids, fatty acids, amino acids, proteins, nucleosides, phenols, alkaloids, and inorganic elements [12]. Among them, GL polysaccharides (GLP) and triterpenes (GLTs) have become their main bioactive ingredients due to their high content, diverse structure, and significant biological activity [13,14]. Therefore, it is necessary to separately discuss these two main components. Modern pharmacological research shows that the bioactive ingredients such as polysaccharides and triterpenes enable GL to have anti-tumor, anti-inflammatory, hypoglycemic effects. Notably, GL has shown significant clinical efficacy in the treatment of conditions such as diabetes, liver and kidney diseases, as well as malignant tumors. Fig. 2 summarizes the main active ingredients and related pharmacological effects of GL.

**Table 1:** Introduction to proximate composition of the fruiting body of GL from different countries or regions, adapted from [15,16].

Constitutes/Origin	Fibers %	Carbohydrates %	Proteins %	Lipids/Fats %	Ash %	References
**China**	76.81 ± 3.46	9.88 ± 1.04	7.47 ± 0.22	–	1.21 ± 0.06	[17]
**Taiwan**	59	26–28	7–8	3–5	1.8	[18]
**Europe(g/100g)**	–	82.3g/100g	13.3g/100g	3.0g/100g	–	[19]
**India**	–	42.8	23.6	5.8	18.7	[20]
**Pakistan**	54.1	82.47	15.04	0.53	2.01	[21]
**Bangladesh**	14.67	44.91	28.6	2.4	3.93	[22]
**Spain**	69.35 ± 3.12	11.02 ± 1.16	11.70 ± 0.35	–	2.31 ± 0.12	[17]
**Nigeria**	3–32	3–28	10–40	2–8	8–10	[23,24]

### 2.2 GLP

GLP has multiple pharmacological activities, with various pharmacological effects, especially significant effects in immune regulation and anti-cancer. In recent years, most studies have focused on the extraction, purification, and structural characterization of GLP from its fruiting bodies, spores, mycelium, and culture media [27].

#### 2.2.1 Extraction and separation of GLP

**Fig. 3:**
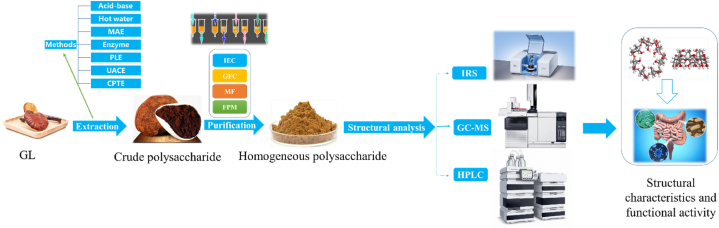
Flow chart of extraction, purification and structural characterization analysis of GLP [25,37].

GLP is a water-soluble polysaccharide. Currently, research has discovered over 200 different polysaccharides from GL, including α-D-mannan, β-D-glucan, α-D-glucan and polysaccharide protein complexes, etc [28]. The extraction and separation process of GL involves [29,30]: the extraction of crude polysaccharides, separation and purification. The main methods include ethanol precipitation [31], microwave-assisted extraction (MAE) [32], enzyme extraction [33], membrane filtration (MF), fractional precipitation method (FPM) and other methods [29,30]. The extraction and purification process and structural characterization analysis diagram are shown in Fig. 3.

Different extraction and separation processes for GL will have different effects. Z. S et al. proposed an ultrasound assisted co extraction (UACE) process that the antioxidant capacity of the obtained polysaccharides is higher than that of traditional hot water extraction methods [34]. In a study using response surface methodology to optimize ultrasound assisted enzymatic extraction (UAEE) for extracting polysaccharides from Vietnamese Red GL, it was found that the highest content of polysaccharides in the extraction solution was 32.08 mg/g [33]. Comparison of MAE and pressurized liquid extraction (PLE) for extracting GLP revealed that time had a certain effect on MAE but had no effect on PLE. Both extraction methods were effective in extracting GLP [35]. The continuous phase change extraction (CPTE) method causes damage to most cells, effectively releasing intracellular components and significantly increasing the yield of GLP, which is 3.34 times and 2.68 times higher than that of hot water extraction method and UACE, respectively [36]. Ethanol precipitation method eliminates impurities in polysaccharide extraction, and other technologies, including fractionation precipitation method, acetic acid precipitation method, ion exchange chromatography, gel filtration method and affinity chromatography, can successfully obtain high-quality polysaccharides from GL [37]. Overall, the UACE + ethanol precipitation process is more economically viable extraction and purification method at present.

At present, most research on the extraction of GLP mainly focuses on the crude polysaccharide stage, and many reports show that crude polysaccharides are directly used for the treatment and prevention of various diseases. In fact, purifying crude polysaccharides to separate various types and studying their structural characterization will further promote the application of GLP in the field of human health.

#### 2.2.2 The structural characterization of GLP

The biological activity of polysaccharides is related to their complex structure, including molecular weight, monosaccharide composition, glycosidic bonds, and substituents. Analyzing the structure of polysaccharides is the basis for establishing the relationship between polysaccharide structure and biological activity [[38], [39], [40]]. At present, the main determination techniques for GLP structure include infrared spectroscopy (IRS) [41], GC-MS [42], HPLC [43], etc. S. Fr et al. used GC-MS to analyze the monosaccharide composition of GLP under optimal conditions, and the results showed that the extract of GL was mainly composed of glucose and exceeded 86 %, confirming that the GL extract was rich in glucan polysaccharides [35] (Table 2).

**Table 2:** Monosaccharide composition of the extracts obtained from GL, adapted from [35,40].

Rrigin	Extraction methods	Monosaccharides (%)^a^		
		Glucose	Mannose	Galactose
GL	MAE	89.6	7.1	1.1
	PLE	86.5	9.4	1.8

a Alditol acetates obtained on successive hydrolysis, NaBH4reduction, and acety-lation.

The sugar components of GLP are mainly glucose, mannose, rhamnose and galactose, may be pure polysaccharides or polymers synthesized by binding with other proteins or peptides, and the molecular weight of polysaccharides in different parts has obvious differences [44]. In addition, the glycosidic bonds of the GLP backbone are formed by α- (1 → 3) dextran α- (1 → 6) glucan, mannan and galactosan are composed of single or mixed bonds, and have α, β- Dextran or other bonds [45]. The studies of AHMAD et al. [39] and Liu Yanfang et al. [46]indicate that the active polysaccharides isolated from GL mainly include β (1 → 3), β (1 → 4), and β (1–6) - D-glucan, mainly composed of β-glucan, isopolysaccharide, and glycoprotein. A novel homogeneous heteropolysaccharide GSPB70-S isolated from GL is mainly composed of Glc, GlcN, etc [47]. Water soluble GL polysaccharides (WGLP) only contain β- D-Glc residue, main chain is composed of (1 → 3)- β-d-glucuronic acid residues, the side chain is composed of the end and (1 → 6)- β-d-glucuronic acid residues and connected at O-6 position [48,49].

GLP has high molecular weight β- (1 → 3) - glucan structure [50], has a relatively high molecular weight and diverse monosaccharide composition, which is closely related to its immunomodulatory and antitumor effects [45,51]. Research has found that using cassava straw as a carbon source for GL can effectively increase the molecular weight of GLP[52]. However, because the structure of GLP is complex and diverse, its function is the result of the interaction of multiple factors, rather than relying on a single influencing factor. For example, the activity of β- (1 → 3) - glucan is strongly dependent on high molecular weight, while super high molecular weight and branched polysaccharides are difficult to trigger immune responses through receptor recognition patterns, but they can be used as prebiotics in vivo [[53], [54], [55]]. Therefore, how to balance the structure and functional activities of Glycochemistry in practical research and application is still a huge challenge for the current scientific community. Meanwhile, for a long time, the standardization of polysaccharide structure analysis and detection techniques has been a challenge in the industry. Due to the lack of unified standards for chromatographic analysis and structural characterization, polysaccharide structures measured from the same raw materials and extraction methods may differ [56]. Therefore, for the analysis of the structure-activity relationship of GLP, it is necessary to focus on the key structures that can control polysaccharide activity, and use multiple analytical techniques to combine, gradually improving the level of technical standardization to avoid the influence of a single factor on the results.

### 2.3 GLTs

The chemical structure of Ganoderma triterpenes (GLTs) is relatively complex, with a relative molecular weight generally between 400 and 600. They are highly lipid soluble and poorly soluble in water [57]. GLTs include GAs, Ganoderma glycol, Ganoderma aldehyde, Ganoderma diacid, Ganoderma sterone, and Ganoderma triol. Among them, GAs are the most famous triterpenoids in the Ganoderma genus fungi [58], with complex structures, high molecular weight, and high lipophilicity. They are highly oxidative derivatives of lanosterol [59].

#### 2.3.1 Extraction and analysis of GLTs

GLTs isolated from the fruiting bodies, cultured mycelia and basidiospores of GL belong to trichostane triterpenoids [60]. Different extraction and analysis methods can effectively identify different types of triterpenoid compounds [61]. The comparison of chemical fingerprint spectra and chemometric analysis of GL can reveal the correlation between its chemical spectra and anti proliferative activity, providing good guidance for studying the relationship between chemical spectra and biological activity of GL [62]. Wang Jiajia et al. used ethanol extraction method to obtain the crude alcohol extract of GL, further extracted with ethyl acetate, chloroform acetone and chloroform methanol gradient elution to obtain GLTs compounds, and then detected by GC-MS that the main compounds were 2α,3α,23-trihydroxy-urs-12-en-28-oic acid [63]. Zhou et al. extracted and isolated two triterpenoids from the fruiting bodies of GL in Australia: Ganoastralins A(1) and B (2), which opened up a new way for the potential of GLTs production [64]. Veena Ravindran K. et al. used high-performance thin-layer chromatography to determine the chemical spectrum of total triterpenes in GL and found that GLTs have potential therapeutic value in improving doxorubicin (DOX) induced cardiomyopathy in rats [65]. Huang XiuRun et al. isolated a new triterpenoid and three known compounds from GL fruiting body using silica gel column chromatography and Sephadex LH-20 column chromatography, were analyzed using nuclear magnetic resonance and mass spectrometry to determine their structures, achieving good results [66]. Comparing the triterpenoid derivatives of five GL related species produced in Vietnam with GLTs from other Asian countries such as Japan, South Korea, and China using high pressure thin-layer chromatography (HPTLC), provides an important basis for identifying GL varieties in Vietnam and other Asian countries [67]. For now, ethanol extraction is the more economical method, while HPTLC will be more promising in the future.

#### 2.3.2 The structural characterization of GLTs

The current synthetic structure of GLTs is derived from lanosterol, and its skeleton is a tetracyclic structure with the molecular formula of C_30_H_48_ [68]. The terpenoids in GL, characterized by isoprene units and having a C30 skeleton structure of ganoderic acid, aldehydes, esters, alcohols, lactones, glycosides, and ketones, with molecular weights ranging from 400 to 600 kDa, are mainly composed of lanosterol carbon skeletons and pentacyclic triterpenes [69]. Wu et al. reported that GLTs extracted from mycelia, fruiting bodies and spores of GL showed obvious structural changes, including alcohols, aldehydes, ketones, acids, esters and other substituents at different positions [70]. C ö R et al. and Lin et al. further studied the structure and characterization of triterpenoids in GL, proposed the molecular configuration of GAs, and clarified that GAs have biological activities such as anti-tumor, antibacterial, regulating immunity, delaying aging [5,57].

In addition, in-depth research on the structure and function of secondary metabolites of GLTs will lay the foundation for establishing the important pharmacological effects of GL [71]. Koo et al. isolated a new Ganoderma ketone a (1) and eight known derivatives (2–9) from the methanol extract of GL, and found that the structure of the compound was related to the inhibitory effect of lipopolysaccharide (LPS) induced RAW 264.7 NO production in mouse macrophages, at the concentration of 50 μM, compounds 4 and 7 exhibit an inhibitory effect on NO of 86.5 % and 88.2 %, respectively [72]. Li Da Wei et al. isolated 29 triterpenes from ethanol extracts of GL fruiting bodies, and structural characterization revealed that within the 100 μM range, the inhibitory effect of all triterpenes on fatty acid amide hydrolase (FAAH) ranges from 30 % to 60 %. This study provides evidence of Ganoderma as a functional food or drug supplement for the treatment of neuroinflammation [73]. The specific structure of GLTs is closely related to the pharmacological mechanisms of lowering blood pressure, lowering blood lipids, and protecting liver, and the synthetic compounds produced by structural modification of GLTs may have significant activity against *virulent strains* (H37Rv) and extensively drug-resistant *tuberculosis strains* (H37Ra) [74,75].

## 3 The effects of GL on human health

**Fig. 4:**
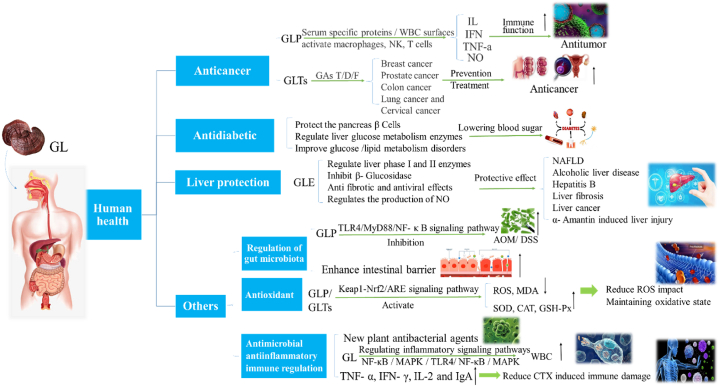
The effects of GL on human health.

Whether used alone or in combination with drugs, GL can serve as a functional food, nutritional supplement, or adjuvant in modern medicine, exerting positive and beneficial effects on human health. However, there is still much to be discovered regarding the underlying mechanisms through which GL impacts human health.Therefore, exploration in this field remains a key focus for the future (Fig. 4).

### 3.1 Anticancer properties

GL has become a new approach to the treatment and prevention of cancer using traditional and modern combination therapies, as it is rich in natural active ingredients such as GLP, GA-t, GA-dm, GA-h, GA-f, GA- γ and GA - me [76]. Polysaccharides and triterpenes have received attention due to their beneficial evidence in preclinical and clinical anti-cancer research.

GLTs also have significant effects in the prevention and treatment of different types of cancer, such as breast cancer, prostate cancer, colon cancer, lung cancer and cervical cancer. Various clinical and preclinical trials have shown that different types of triterpenes, such as ganoderic acid T, ganoderic acid D, and ganoderol F, have significant anti-cancer effects, especially ganoderic acid (GAs), which contain several important pharmacological and therapeutic properties for human diseases and are the main source of new drug development [77]. Satria Dedi et al. isolated a novel triterpenoid ganoderic acid D (1) (lucidomol D) from the fruiting body of GL, and detected its cytotoxicity on several tumor cells using MTT assay. The results showed that lucidomol D has selective anti proliferative and cytotoxic effects on MCF-7, HepG2, HeLa, Caco-2, and HCT-116 [59]. Li et al. and Jiao et al. studied the anticancer effects of neutral triterpenoid fraction (NTF) and Ganoderma spore oil (GLSO) extracted from GL, respectively. The results showed that NTF is a potential anticancer drug for colon cancer, and its active ingredient may be Ganoderma alcohol; GLSO inhibits the growth of MDA-MB-231- cells and tumors by inducing cell apoptosis in vivo, indicating that the anticancer mechanism of GL may be related to the activation of mitochondrial dependent pathways [78,79]. Wang et al. investigated the mechanism of action of GL on prostate cancer (PC-3) cells by influencing the JAK-1/STAT-3 signaling pathway. They found that GL inhibits the development and apoptosis of prostate cells by inhibiting STAT-3 translocation, and has a significant cytotoxic, ROS increasing, and apoptotic stimulating effect on PC-3 cells [80]. GLTs can improve the therapeutic effect of colorectal cancer by inhibiting NF-kb regulated inflammation and carcinogenic mechanisms [81]. GLTs can reduce the incidence and diversity of skin tumors, significantly reverse UV mediated DNFB induced CHS inhibition, and their prevention of skin cancer may be achieved by weakening UV induced immunosuppression [82].

The enhanced immune function mediated by GLP is the core mechanism of GL's anti-tumor effect, mainly by binding to serum specific proteins or leukocyte surfaces, activating effector cells such as macrophages, natural killer cells (NK), helper T cells, and increasing the production of cytokines such as IL, interferon (IFN), TNF-a, nitrogen monoxide (NO), and antibodies, thereby enhancing the host's defense and producing anti-tumor effects [83]. GL can inhibit the growth of K562 leukemia cells and stimulate their differentiation into more mature red blood cells, and improving immune function in mice [84]. Guo Cuiling et al. found that GLP reduced AOM/DSS induced colitis and tumor development, manifested by a significant decrease in disease activity index score, total number and size of tumors, making it a promising prebiotic for the treatment of colorectal cancer [85]. GLP exhibits anti-cancer effects through various mechanisms, including cytotoxicity, antioxidant properties, induction of cell apoptosis, production of ROS, and anti proliferative effects. Nanoparticles (NPs) based on GLP have the potential to be designed as biologically active ingredient delivery carriers for various cancer tissues, representing an emerging and promising pathway for inhibiting cancer and reducing chemotherapy side effects, promoting the application of GL as a natural adjuvant drug in combination therapy [86].

Furthermore, as an adjuvant drug in cancer treatment, there is potential in exploring the synergistic effects between active ingredients of GL and chemotherapy drugs, which can lead to improved cancer treatment outcomes [14,87]. Additionally, certain derivatives of GL, despite exhibiting some level of toxicity, have garnered attention in experimental studies and clinical practice [88]. However, the utilization of GL as a primary cancer treatment drug is limited. The main hindrance in its clinical research lies in the absence of purified compounds or precisely characterized extracts, which can be attributed to variations in growth conditions, strains, and extraction techniques. In order to address this issue, future clinical investigations should emphasize the assessment and chemical characterization of specific compounds within GL. Therefore, this article summarizes and compares the global standard status of the GL industry, which can provide a suggestion for future industry standards. This will facilitate the determination of precise dosages for clinical application.

### 3.2 Antidiabetic properties

Research has shown that the main active components of GL, GLP and GLTs, have a good therapeutic effect on diabetes. Their main functions are to regulate liver glucose metabolism enzymes, improve glucose and lipid metabolism disorders, protect the pancreas β Cells, and regulate the expression of oxidative stress-related factors and pathways, thereby preventing hyperglycemia [89].

Ekiz Elif et al. found that GLP and GLTs have potential benefits in the treatment of metabolic disorders such as diabetes and obesity, which can improve insulin sensitivity and reduce blood sugar levels in diabetes animal models, and GLP also has the effect of reducing weight and improving glucose metabolism [90]. Yao Xiaolin et al. studied the effect and mechanism of GLP on diabetes induced erectile dysfunction (DMED) (a common complication of diabetes), and used low dose (GLP l)/high dose (GLP h) of GLP to treat DMED rats respectively. The results showed that GLP can effectively improve DMED by repairing CC pathological damage, up regulating NOS expression and ERK/JNK phosphorylation, indicating that GLP may be a candidate drug for treating DMED [91]. GLP can promote the expression of Nrf 2 and HO^−1^, regulate the expression of oxidative stress-related factors and pathways, and thus play a role in reducing blood sugar [92]. Yang et al. found that a new protein polysaccharide Fudan Yueyang G. clucidum (FYGL) extracted from GL inhibits the expression of protein tyrosine phosphatase 1B (PTP1B) at the mRNA level, phosphorylates insulin receptor substrate 1 (IRS1), promotes the plasma membrane transport of glucose transporter type 4 (GLUT4), and achieves hypoglycemic effects [93]. In obese diabetes, GLP balances adipogenesis and lipid metabolism, regulates metabolic disorder and unhealthy obesity, and can be used as a promising drug to treat metabolic obesity [94].

Type 2 diabetes (T2DM) is a long-term metabolic disease caused by genetic and environmental factors. Obesity is often accompanied by metabolic disorder and insulin resistance, leading to type 2 diabetes [95]. Xiao et al. used type 2 diabetes mice to evaluate the anti diabetic effect of four polysaccharide rich components (F21, F22, F31, and F32) extracted from GLP. The results showed that F31 significantly reduced FSG (fasting saliva glucose), FSI (fasting serum insulin), and epididymal fat/body weight ratio (p0.05, p0.01), indicating that F31 is the main bioactive substance of GLP [96]. Pan Rui et al. treated T2DM mice with GLP (100 and 400 mg/kg) for 8 weeks. The results showed that GLP could significantly improve the imbalance level of diabetes related indicators in T2DM mice, reduce blood glucose concentration, promote insulin secretion, and improve glucose tolerance [97]. Khursheed Rubiya et al. found that the composition containing GL extract had a significant effect on reducing blood sugar in streptozotocin induced type 2 diabetes rats [98].

Traditional treatments for diabetes have limitations, including adverse side effects and high costs associated with continuous care [99]. GL has demonstrated significant potential in reducing the adverse effects associated with traditional diabetes treatment methods and lowering healthcare expenses.so it is crucial to further investigate the profound impact of GL on diabetes.

### 3.3 Liver protective properties

GL exhibits a wide range of protective effects in various liver diseases, such as non-alcoholic fatty liver disease (NAFLD), alcoholic liver disease, hepatitis B, liver fibrosis, liver cancer, and carbon tetrachloride (CCl4) α- Amantin induced liver injury, etc [100].

Ganoderma lucidum extract (GLE) has a strong preventive and protective therapeutic effect on formaldehyde (FA) induced liver fibrosis [30,101]. Yue Leng et al. found that there were differences in the hepatoprotective effects of GL spore powder and unbroken spore powder on acute alcoholic liver injury in mice. Both products significantly reduced serum levels of aspartate aminotransferase (AST) and alanine aminotransferase (ALT), as well as the release of inflammatory factors, effectively improving the pathological state of liver cells. However, the effect of wall breaking products was better [102]. Ganoderma acid polysaccharide (GAP) can effectively delay the onset of NAFLD, while reducing blood lipid levels, liver weight, and liver quality. It can be used as an effective preventive and therapeutic drug for NAFLD [103]. A study on the toxic effects of Mexican GL extract on the liver and kidneys of Wistar rats, indicating that GL extract had no significant adverse effects on the liver of male and female rats [104]. Antunes et al. investigated the effects of GL fruiting body dry extract (GLE) on human liver (HepG2/C3A) and kidney (786-O) tumor cells and peripheral blood lymphocytes. The results showed that GLE exhibited potential anti-tumor effects on cancerous kidney and liver cells, exhibiting cytotoxic and genotoxic activities at low concentrations [105]. GAs have a good protective effect on alcoholic liver injury in mice [106]. Lv et al. found through research that GL acid (GA)-a has a significant regulatory effect on the composition of liver metabolites in alcohol exposed mice. GA-a intervention controlled the mRNA levels of genes involved in alcohol metabolism, bile production, oxidative stress, and other substance metabolism in the liver, significantly reducing the impact of alcohol poisoning on the liver. It has the potential to be developed as a new type of nutrient for preventing alcohol poisoning [107].

However, co ingestion of GL with other substances may also lead to varying degrees of liver toxicity. The study by Guedikian Roupen et al. reported a 47 year old male case who experienced headaches and abdominal pain after taking GL powder and alcohol. He was diagnosed with acute hepatitis with obvious transammonitis and only improved after 2 weeks of treatment [108]. This indicates that although GL based nutritional supplements are becoming increasingly popular, the potential impact on co intake with other substances such as alcohol remains to be explored.

### 3.4 Other effects on human health

#### 3.4.1 Regulatory properties of gut microbiota

Research has demonstrated that GL confers health benefits on human gut microbiota by maintaining intestinal homeostasis through promoting the growth and metabolism of gut probiotics, and by improving intestinal metabolism to lower blood sugar and blood lipids [54,104]. GLP extracted from dewalled Ganoderma spores improved AOM/DSS induced microbial dysbiosis and significantly improved intestinal barrier function by inhibiting the TLR4/MyD88/NF-κB signaling pathway [79]. Ganoderma lucidum ethanol extract (GLE), as a prebiotic food extract, combined with ciprofloxacin in the treatment of *Salmonella* infection, can reduce the side effects of clinical use of antibiotics alone, increase the number of probiotics in the intestine [109,110]. The active ingredient Ganoderma lactone B in GLTs can improve gut microbiota imbalance and inhibit the development of non hepatitis B virus (NHBV) associated liver cancer (HCC) [111]. Ganoderma spore polysaccharide (GLSP) can alleviate the imbalance of gut microbiota, and broken GLSP has a better effect than unbroken GLSP [30].

These findings suggest that strategies to alter gut microbiota and maintain basic physiological parameters through GL nutritional supplements may be a useful approach. However, currently, most reports on the health regulation of gut microbiota by GL are based on in vitro and in vivo animal studies, with limited research based on clinical trials. In addition, the metabolic recognition patterns and pathways of GL extract in the intestine still need to be verified and explored.

#### 3.4.2 Antioxidant properties

GL is a natural antioxidant raw material with no toxic side effects, which is used for the combined treatment of many diseases. It can protect tissues from the toxicity of ROS, maintain the body's oxidative state, and reduce patient adverse reactions [112,113]. The study on the antioxidant capacity of cultivated and wild GL by supercritical carbon dioxide (SCCO2) extraction found that cultivated samples have stronger antioxidant capacity than wild samples [114]. GL also contains a large amount of flavonoids and phenolic compounds, and the fermented Ganoderma spore powder polysaccharides have a more significant role in antioxidant regulation [[115], [116], [117]]. Ş. Canpolat et al. used the DPPH radical scavenging method to determine the antioxidant activity of natural GL methanol extract, indicating that GL has strong antioxidant activity [118]. Mousavi Roghieh Sadat et al. investigated the effect of adding GL extract on the total phenolic content and antioxidant capacity of UF white cheese during fermentation. The results showed that the total phenolic content and antioxidant activity were significantly increased, and the UF white cheese with 1.0 % GL added had the best quality indicators [119].

#### 3.4.3 Antimicrobial, antiinflammatory and immune regulatory properties

GL extract has great market potential as a new source of antimicrobial agents. S á nchez Hern á ndez Eva et al. found that GL extract has anti egg and anti bacterial activity against *Phytophthora cinnamomi* and three *Botryosphaeriaceae fungi*, with in vitro inhibitory concentrations (MICs) of 187.5 μg mL^−1^ and 187.5–1000 μg mL^−1^, respectively, the MIC value of this study is one of the highest reported natural product MIC values against plant pathogens to date [120]. Moroccan GL extract exhibits effective antibacterial properties against 7 human pathogenic microorganisms in the concentration range of 1–16 mg/mL, with the most sensitive being the flocculent epidermal bacteria (MIC = MFC = 1 mg/mL) and the most resistant being *Aspergillus fumigatus* (MIC = 16 mg/mL, MFC ≥16 mg/mL). It can be used as a potential PET (positron emission tomography) tracer for studying inflammation, There is enormous potential for application in the food and pharmaceutical industry [114]. Ş. Campolat et al. studied the inhibitory effects of natural GL methanol and acetone extracts on pathogens and found that acetone extracts had the highest antibacterial activity against *Enterococcus faecalis* ATCC 29212 [117].

GL also has strong antiinflammatory and immune regulatory functions. GLP inhibits TLR4/NF-κB and mitogen activated protein kinase (MAPK) inflammatory signaling pathways regulate tumor necrosis factor-α(TNF-α), nitric oxide (NO), and the levels of inflammatory factors such as Interleukin (IL) −6, IL-1β, IL-18, and IL-10 to inhibit the production and expression of proinflammatory factors, thus playing an anti-inflammatory role [91,121]. In addition, GLP can activate silent information regulator 1 (SIRT1), regulate inflammatory response, and inhibit cell apoptosis [122]. GL polysaccharide peptide (GLPP) improves the immunosuppressive effect induced by cyclophosphamide (CTX), and 100 mg/kg/d GLPP can significantly alleviate CTX induced immune damage by improving the immune organ index, cytokine (TNF-α, IFN-γ, IL-2) and immunoglobulin A (IgA) secretion in mice, and compared to unfermented GL, fermented GL has a better effect on intestinal immunity [123,124].

Currently, the development speed of nutritional and functional products worldwide is relatively fast, and the demand for GL nutritional and health foods from consumers continues to increase. As more scientific research shows the potential benefits of GL for human health, how to scientifically and reasonably develop products and promote industrial applications will be one of the current and future key directions in this field.

## 4 Industrial standardization of GL

Under the backdrop of economic globalization, nutrition and health have become universal topics that transcend national boundaries. Countries around the world place immense value on the nutritional value and safety of traded food and pharmaceuticals [125]. In recent times, the rapid advancement of novel food and pharmaceutical processing technologies has seen the industrial application and development of GL. This has effectively addressed the shortcomings of traditional cultivation and processing methods, including their lengthy cycles, low yields, and inefficient nutrient utilization. GL has thus played a crucial role in safeguarding the populace's health, ensuring their well-being, and fostering economic and social progress. Standardization emerges as a key element and a prerequisite for the rapid evolution of the GL industry. The global health sector's standardization level is improving continually. This process aids in enhancing product quality and safety while promoting standardized management within the industry, consequently contributing to its orderly development. To facilitate further growth in this sector, it is imperative to establish and refine pertinent international standards, thereby laying a robust foundation for the GL industry's future advancements.

### 4.1 The whole industrial chain of GL

GL has enormous market and industrial potential. According to data, China is the world's largest producer of GL, and the Ganoderma industry in Japan and South Korea is also significant, Japan consumes about 1500 tons of GL annually, while South Korea consumes around 4500 tons of GL annually [12]. Currently, the standardized development of the whole industry chain of GL, from cultivation, production, processing to sales and circulation, is accelerating. The standardized application of some novel quality control techniques, such as isotope ratio mass spectrometry (IRMS), which can characterize GL according to species, region of origin and cultivation method, will provide valuable insights into controlling fraudulent labeling of GL and support regulatory agencies in authenticating claims of origin [10]. The consumer market for tea, alcoholic beverages, and nutritional health products has reached billions of dollars, providing thousands of products to the market [12,126]. The standardization of the determination and analysis of GL functional active ingredients such as GLP and GLTs has become increasingly important. The market credibility and quality of GL products are constantly improving, and the industrial advantages and huge market demand of GL make the standardization demand urgent.

**Fig. 5:**
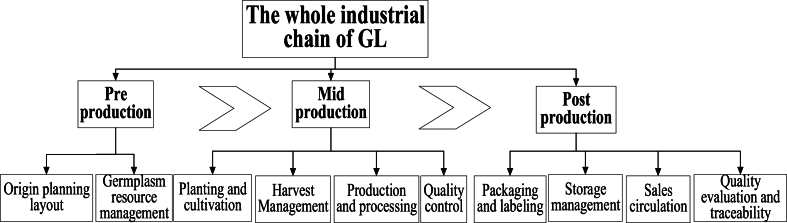
The whole industrial chain of GL.

**Fig. 6:**
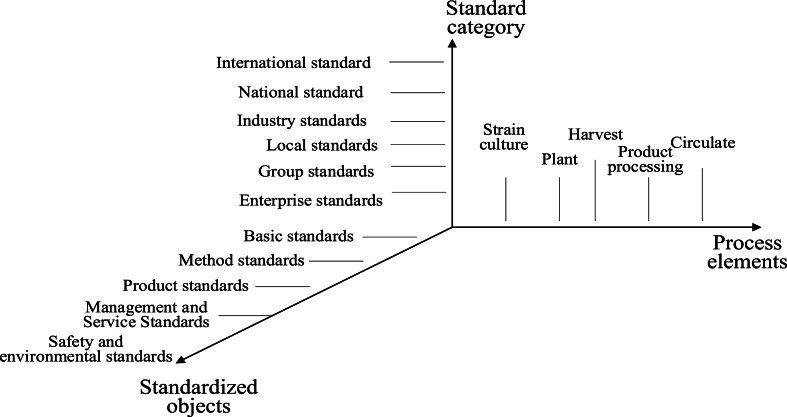
A three-dimensional framework model of the standard system for the entire industry chain of GL.

**Fig. 7:**
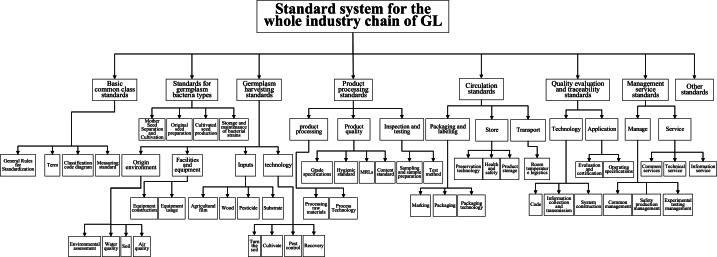
Structure diagram of the standard system framework for the entire GL industry chain.

This article presents a schematic diagram of the entire industry chain of GL in three stages: pre production, mid production, and post production (Fig. 5). Pre production mainly includes the planning and layout of production areas and the management of germplasm resources; Mid production includes planting and cultivation, harvesting management, production processing, and quality control; Post production includes packaging labeling, storage management, sales and circulation, quality evaluation and traceability [127]. In addition, a three-dimensional framework model of the standard system for the entire GL industry chain was created from three different dimensions: standard categories, standardized objects, and process elements (Fig. 6). On this basis, a framework diagram of the GL industry chain standard system was further organized and formed (Fig. 7), which mainly includes 8 types of standards, namely basic common standards, germplasm and mushroom type standards, planting and harvesting standards, product processing standards, circulation standards, quality evaluation and traceability standards, management and service standards, and other standards [128]. Specific standard directions were proposed to provide reference and inspiration for the standardization construction of the GL industry.

### 4.2 The status quo of domestic and foreign standards for GL

GL originated in China, and the United States, Europe, Japan, and South Korea are the main markets for GL consumption throughout the year. Although further exploration of the active mechanism of GL on human health is needed, countries and international organizations around the world have established corresponding standards for the use of GL.

#### 4.2.1 International organizations

Internationally, in 2009, the International Organization for Standardization (ISO) officially established the Technical Committee for Traditional Chinese Medicine (ISO/TC 249), which immediately became the main platform for the formulation, implementation, and promotion of international standards for Chinese medicinal materials such as GL. In 2019, the official website of the ISO/TC 249 officially released the international standard for GL “Traditional Chinese medicine — *Ganoderma lucidum* fruiting body” (ISO 21315: 2018) [129], which was developed by China Shouxiangu Company. This is the third quality standard for traditional Chinese medicine released by ISO/TC 249, and also the world's first international standard for GL. This standard mainly regulates the quality standards, testing indicators, testing methods, packaging, storage, transportation requirements, etc. Clarifies that GLP and Ganoderic acid A are iconic components of GL, and provides corresponding testing methods, providing a unified standard for international trade in the GL industry and reducing technical trade barriers to industry internationalization. This not only lays a solid foundation for China's traditional GL industry to go global, but also a major breakthrough in the internationalization of traditional Chinese medicine. In addition, the ISO international standard on ganoderma lucidum spore powder has been under development. ISO has also developed the “Cultivated Mushrooms Guide to cold storage and refrigerated transportation first edition” (ISO 7561:1984) [130], which provides guidance on the refrigeration and refrigerated transportation.

GL belongs to the genus of edible mushrooms. The Codex Alimentarius Commission (CAC) does not have a specific standard for GL. However, two edible mushroom related standards have been developed, namely the Codex General Standard for Edible Fungi and Fungus Products (CodexStan 38–1981) [131]and the Codex Standard for Dried Fungi (Codex Stan 39–1981) [132]. There are relevant regulations on the quality of edible mushroom related products and raw materials, and some clauses in the above two general technical standards are applicable to GL products.

The international standards related to GL provide important support for the high-quality development of the industry and international trade, and the ISO or CAC standards are also important references for the development of standards in countries around the world.

#### 4.2.2 China

China is the birthplace of Ganoderma culture and a major exporter of Ganoderma cultivation and raw materials. In recent years, China has done a lot of work in the standardization of the GL industry, continuously promoting traditional Chinese medicinal materials such as the GL industry to go global. Given China's status as the largest global producer of GL and its extensive historical consumption spanning over 2000 years, the National Health Commission of China and the State Administration for Market Regulation have officially designated GL as a substance falling under the category of “food and medicine homologous substances” recently. This recognition acknowledges GL's dual role in traditional Chinese medicine and as a consumable product. Consequently, this significant milestone is projected to substantially propel the industrialization of GL in both the food and medicinal sectors [133,134].

In the 2020 edition of the Chinese Pharmacopoeia (ChP, 2020 vol 1) [135], Ganoderma spore powder is listed as a type of traditional Chinese medicine, belonging to the family of porous fungi, *Ganoderma lucid* um (Leyss. exFr.)Karst. or the dried fruiting body of *Ganoderma sinense*. It specifies the quality standards, identification, content determination, microbial limits, and other requirements for GL spore powder. The pharmacopoeia provides regulations for the detection and content of the main active ingredients of GL, including GLP, triterpenes, and sterols. GLP, calculated as anhydrous glucose (C_6_H_12_O_6_), shall not be less than 0.90 % of GL dried products, while triterpenes and sterols, calculated as oleanolic acid (C_30_H_48_O_3_), shall not be less than 0.50 % of GL dried products. These regulations and standards provide guidance and assurance for the production and quality control of Ganoderma spore powder.

In addition, the Chinese national standard “Technical specifications for harvesting and processing of Ganoderma spore powder” (GB/T 29344-2023) [136] also provides specifications for the harvesting and processing technology of Ganoderma spore powder; Mushroom strain (GB/T 19170-2003) [137]specifies the relevant quality and technical requirements for shiitake mushroom strains; The National Food Safety Standards for Edible Fungi and Products (GB 7096-2014) [138]and the Selenium Enriched Edible Fungi Powder (GB 1903.22–2016) [139] have made corresponding provisions on the food safety requirements for edible mushrooms and their products.

In terms of industry standards formulated by Chinese government departments, the National Medical Products Administration of China has stipulated the quality requirements for GL (Chizhi) formula granules (YBZ-PFKL-2021086) [140]; The Ministry of Agriculture and Rural Affairs has also formulated relevant standards, “Determination of wall breaking rate of GL spore powder” (NY/T 1677–2008) [141]specifies the method for determining the wall breaking rate of GL spore powder; “Determination of GL acid content in Ganoderma products by high-performance liquid chromatography” (NY/T 2278-2012) [142]specifies the determination of GL acid content in Ganoderma products by HPLC method; The determination of total triterpenoid content in Ganoderma lucidum by spectrophotometry (NY/T 3676-2020) [143]is specified.

In addition to the above, China has also formulated large number of local standards and group standards, which together have made significant contributions to the standardization and internationalization development of the Ganoderma industry.

#### 4.2.3 USA

The United States Pharmacopoeia (USP) is an internationally recognized authoritative organization in the pharmaceutical and medical fields. Among them, the American Herbal Pharmacopoeia and Therapeutical Compendium (AHPT) is a specialized collection of medicinal plants available for market circulation, and is the most important basis for the operation and use of herbal products in the United States, with recognized authority in the international community. In November 2010, after being reviewed by the US FDA, GL became one of the first five traditional Chinese medicines to enter AHPT. This means that GL, which has been used for over 2000 years, has entered a new global stage [144].

In the United States Pharmacopoeia (USP-NF 2022), Ganoderma spore powder is listed as a natural medicinal herb and widely used in various research and development. The US Pharmacopoeia sets quality standards and testing methods for Ganoderma spore powder, including indicators such as appearance, content, microbial limit, heavy metals, etc. The US Pharmacopoeia (USP38-NF33) [145]also strictly requires that the effective ingredient content of Ganoderma spore powder should not be less than 1.5 %, GLP should not be less than 0.7 %, and Ganoderic acid A should not be less than 0.3 %. Among them, the standard for measuring GLP content based on HPLC in the United States Pharmacopoeia is widely used worldwide. This method uses mannose, D-glucuronic acid, galactose RS, glucose, and L-fucose as standard substances, and uses high-performance liquid chromatography to detect and calculate their total polysaccharide content. The advantages of this method are high accuracy, high sensitivity, and good reproducibility. But there are also some shortcomings, such as issues in sample pretreatment and sensitivity. For GLTs, the US Pharmacopoeia stipulates that the content of triterpenoids in GL Fruiting Body is calculated based on dried GL fruiting body, including Ganoderma A, B, C2, D, F, G, H, and Ganoderic acid B, C, and D. Ultra high performance liquid chromatography (UHPLC) is used to calculate the total content of ten triterpenoids using the conversion factor method, with Ganoderma A as the reference substance, as the content of triterpenoids.

In addition, when GL was used as an edible mushroom product, the United States had established a very complete and scientific system in the inspection and quarantine of import and export commodities. The FDA in the United States uses the Association of Official Analytical Chemists (AOAC) method for the inspection of edible mushrooms: under an off ground microscope, non food organic or inorganic substances such as animal hair (over 0.2 mm), insect fragments, and fine dust are separated, which is referred to as unclean notification.

Overall, since GL was included in the US Pharmacopoeia, it has attracted the attention of US officials and research institutions, and the standardization level in the US market has been increasing, which has played a promoting role in market development.

#### 4.2.4 Eu

The European Pharmacopoeia (EP) is the only guiding document for drug quality testing in Europe. Based on the development momentum of traditional Chinese medicine in Europe, the European Pharmacopoeia set up a special committee on traditional Chinese medicine in 2008 to regulate the sales and use of traditional Chinese medicine and traditional Chinese patent medicines. At present, GL have entered the European Pharmacopoeia through strict testing and verification [146]. If any of the 37 member countries of the European Pharmacopoeia raised questions about GL medicinal materials, they could not be successfully included in the pharmacopoeia. This also means that GL has European recognized standards and specifications in terms of safety, quality, and efficacy, laying the foundation for its wider acceptance and use abroad, and is an important step for GL to open up export channels. GL has been regarded by Europeans as a “magical” herb for delaying aging and assisting in the treatment of various chronic diseases, and is a star herb in the Chinese herbal medicine market.

In addition, as an edible mushroom genus with the same origin as food and medicine, GL is subject to technical regulations and directives such as the European General Food Regulations (Reg EC/178/2002), New Food Management Regulations (Reg EC/258/97), Food Nutrition and Health Claims Regulations (Reg EC/1924/2006), Enhanced Food Management Regulations (Reg EC/1925/2006), and Food Hygiene Regulations (Reg EC/852/2004), which are all applicable to GL in the food industry, it is also an important standard foundation for the export of GL industry to Europe.

#### 4.2.5 Japan

Japan is a major producer and user of plant medicines such as GL. According to statistics, in the global herbal medicine market, Japanese traditional medicine, which is in line with traditional Chinese medicine, holds over 80 % of the market share, while Chinese medicine exports account for about 10 %. In addition, more than 70 % of the production materials for Japanese traditional medicine are imported from China [12]. The dominance of Japanese herbal medicine in the international market is closely related to the standardization. The Japanese Ministry of Health and Welfare (the main department responsible for healthcare and social security in Japan) has established a series of reasonable and strict traditional medicine standards, many of which have been upgraded to international standards and have become a passport for Japanese traditional medicine to go global. In the Japanese Pharmacopoeia, GL is listed as a natural medicinal herb. The more common varieties of GL in Japan include purple GL, gray brown GL, green GL, and GL, which have been promoted to a high level of popularization by the Japanese medical community. The Japanese Pharmacopoeia stipulates the quality standards and testing methods for Ganoderma spore powder, including indicators such as appearance, content, microbial limit, heavy metals, etc. The effective ingredient content of Ganoderma spore powder should not be less than 1.0 %, and its main functions include anti-cancer, immune regulation, and blood sugar lowering, providing important basis for the quality control of Ganoderma spore powder [147].

In addition, Japan's research and utilization of edible mushrooms are also at the forefront of the world. Since World War II, Japan has conducted a significant amount of effective research in the field of edible mushrooms, which has played a significant role in extending the lifespan of its citizens. GL, as an edible mushroom genus, has corresponding detection standards for pesticide residues, heavy metal limits, and so on in the Japanese “affirmative list” system.

The status quo of domestic and foreign standards for GL is detailed in Table 3.

**Table 3:** Domestic and international standards for GL.

Number	Standard category	Standard number	Standard Name	Implementation Date	Remark
1	International Standard	ISO 21315: 2018	Traditional Chinese medicine — Ganoderma lucidum fruiting body	2018/12/20	ISO
2		ISO 7561: 1984	Cultivated mushrooms.Guide to cold storage and refrigerated transport first edition	1984/1/1	ISO
3		CAC/RCP 5-1971	Recommended International Code of Hygienic Practice for Dehydrated Fruits and Vegetables Including Edible Fungi	1971/3/2	CAC
4		CODEX STAN 38–1981	Codex General Standard For Edible Fungi and Fungus Products	1981/6/3	CAC
5		CODEX STAN 39–1981	Codex Standard For Dried Fungi	1981/6/3	CAC
6		CODEX STAN 192–1995	General Codex standards for food additives	1995/6/17	CAC
7	National Standard	GB/T 29344-2023	Technical specifications for harvesting and processing of Ganoderma spore powder	2024/3/1	China
8		GB/T 19170-2003	Mushroom strain		China
9		GB/T 18525.5–2001	Radiation insecticidal and antifungal technology for dried shiitake mushrooms	2001/6/23	China
10		GB 7096–2014	National Food Safety Standards for Edible Fungi and Products	2014/3/1	China
11		GB 2760–2014	National Food Safety Standards for the Use of Food Additives	2014/3/1	China
12		GB 1903.22–2016	National Food Safety Standards Food Nutrient Fortifiers Selenium Enriched Edible Fungi Powder	2016/8/1	China
13		–	pharmacopoeia of the people's republic of china (Part 1, 2020)	2020/5/12	China
14		–	Positive list system	2006/5/29	Japan
15		JP18	The Japanese Pharmacopoeia (JP18)—Crude Drugs and Related Drugs	2022/12/12	Japan
16		USP-NF 2022	American Herbal Pharmacopoeia and Therapeutic Compendium	2022/5/1	US
17		EP11.0	European Pharmacopoeia	2023/1/1	EU
18		Reg EC/178/2002	General Food Regulations	1905/6/24	EU
19		Reg EC/258/97	New Food Management Regulations	2002/6/24	EU
20		RegEC/1924/2006	Regulations on Food Nutrition and Health Claims	2006/5/1	EU
21		Reg EC/1925/2006	Strengthen food management regulations	2006/5/1	EU
22		Dir 2000/13/EC	Food Labeling Directive	2000/3/1	EU
23		Reg EC/852/2004	Food Hygiene Regulations	2004/2/1	EU
24		Reg EC/396/2005	Maximum residue limits for new agricultural and veterinary drugs in food and feed	2005/1/1	EU
25		GOST 34110-2017	Acceptance rules and sampling methods for frozen fruits, vegetables, mushrooms and derived products	2019/1/1	Russia
26		GOST 8756.1–2017	Methods for determining sensory characteristics, composition, and total net weight or volume of fruits, vegetables, and mushroom products	2019/1/1	Russia
27		GOST 13799-2016	Packaging, labeling, transportation, and storage requirements for fruits, vegetables, and mushrooms	2018/1/1	Russia
28		GOST 33318-2015	Specification for dried mushrooms	2017/1/1	Russia
29		GOST R 56636-2015	Specification for fresh cultivated consumption mushrooms	2016/7/1	Russia
30		GOST R 56827-2015	Specification for fresh cultivated mushrooms	2016/7/1	Russia
31		GOST 28322-2014	Terminology and definitions of processed fruits, vegetables, and mushrooms	2015/6/1	Russia
32		GOST R 55465-2013	Specification of frozen mushroom products	2014/7/1	Russia
33		GOST 31916-2012	Mushrooms - Guidelines for refrigerated and refrigerated transportation of mushrooms in cultivated fresh farms	2014/1/1	Russia

### 4.3 The analysis of problems in standardization of GL industry

As can be seen from the above, as a traditional herb, GL has been recognized and promoted in pharmacopoeias in China, the United States, Japan, Europe, and South Korea; As a genus of edible fungi, GL is also included in the management of edible mushroom related standards in various countries. The scope of use and policy regulations of GL may vary in different regions. When using GL in food, the dosage needs to be determined based on its safety assessment results to ensure that its content in food does not have a negative impact on human health.

With the increasing emphasis on health, the rich functional and active ingredients of GL have enormous potential for application in the global health market. In recent years, through in-depth research on key links in the entire industry chain such as breeding, cultivation, and deep processing, the GL industry has gradually ensured the safety, efficiency, stability, and controllability of products. However, the entry of GL into the international health market still requires addressing key issues such as lack of quality standards, poor controllability, unclear active ingredients and mechanisms of action. A comprehensive evaluation of the safety, effectiveness, and quality of GL products has become an inevitable requirement for market development. Therefore, accelerating the standardization construction of the entire GL industry chain has become the focus and key to current industrial development.

In addition, attention should also be paid to the international technical trade barriers faced by the GL industry. Standards have an important voice in international market competition and to some extent represent the initiative in technological and economic competition. With the escalating trend of natural drug research and development abroad, some countries have set up various technical trade barriers under the pretext of safety to protect their interests in the international market. As a traditional Chinese medicine product, GL has also encountered many green barriers, which has brought negative impacts on the global development of the GL industry.

## 5 Conclusion

GL is rich in functional active ingredients with extensive nutritional and pharmacological value. The main active ingredients, such as GLP and GLTs, have complex and diverse chemical structures. Characterization and analysis of the molecular structures of GLP and GLTs remain the basis for exploring the biological activity relationship of GL in the future. At the same time, GL has a wide range of benefits for human health, including anticancer, antidiabetes, liver protection and other aspects (such as regulating gut microbiota, antioxidant, antimicrobial, antiinflammatory and immune regulation). The main manifestation is that it inhibits cancer cell expression through various mechanisms such as cytotoxicity and host immune modulators, and has gradually been used as an adjunctive synergistic therapy for cancer treatment drugs; Reducing blood sugar has obvious effect on reducing side effects of traditional diabetes treatment methods and reducing nursing costs; It has significant protective effects on non-alcoholic fatty liver disease, alcoholic liver disease, and various liver injuries; Promote the growth and metabolism of probiotics in the intestine, improve the gut microbiota, and enhance the intestinal barrier; GL extract is also a non-toxic natural antioxidant, antibacterial and anti-inflammatory agent with significant immune regulatory function. It is used in the combination treatment of many diseases to reduce patient adverse reactions, and is one of the key directions of GL industrialization development. Furthermore, based on the future application of GL in the food and pharmaceutical industry, the article also explores the standardization status of the entire GL industry chain. GL has gradually been included in the pharmacopoeias of multiple countries such as China, the United States, Europe, and Japan. ISO has also established international standards for GL, and the standardization level of the entire GL industry chain from cultivation, production and processing to sales and circulation is gradually improving.

However, at the same time, the reports on the anticancer, antidiabetes and other activities of GL are mostly based on in vitro and in vivo animal studies, while there are few studies based on clinical trials. The metabolic recognition patterns and pathways of GLP in vivo still need to be verified and explored; Some terpenoid derivatives of GL have certain toxicity, and experimental and clinical studies should also be of concern. In addition, the future industrialization development of GL still needs to address key issues such as the lack of quality standards, poor controllability, unclear effective ingredients and mechanisms of action, and accelerate the standard connectivity between global GL industries to reduce green technology barriers.

It can be foreseen that the bioactive value of GL will be more applied in fields such as pharmacy and food health, and the standardization level of the GL industry will gradually develop towards scientific, safety, and sustainable goals with the development of the market, thereby making more contributions to the global health industry and sustainable development. We suggest: Firstly, to speed up the research and exploration of the mechanism of GL's impact on human health. At present, the research exploration in this field is still insufficient, and there are certain contradictions in many different research results; Secondly, the system and content of GL standards, especially international standards, should be dynamically optimised. Standards are the basis and key to ensure food safety, and the content of standard indicators must be dynamically optimised and adjusted along with the development of research results; Thirdly, strengthen the international coordination and cooperation in GL research and standard setting. In order to better assess the biological activity of GL, it is necessary to strengthen international cooperation, and safeguard the rights and health of consumers.

We believe that in the future, with the increasing attention and demand for natural medicinal materials and nutritional health, GL will have broad research and application prospects.

## Funding

The work was funded by Zhejiang Provincial Administration for Market Regulation Science and Technology Plan Project (Project no. ZC2023020).

## Publication ethics statement

The authors of this manuscript affirm their commitment to maintaining the highest ethical standards in accordance with the ethical guidelines provided by Elsevier. This includes adherence to policies on research integrity, publishing ethics, authorship, and the disclosure of potential conflicts of interest.

## Data availability

No data associated with our study has been deposited into a publicly available repository. This study primarily involved the synthesis and analysis of existing literature, and no new experimental data were generated.

No data was used for the research described in the article. This study is based on a comprehensive review and synthesis of existing literature, and therefore, no new data were generated or utilized.

## CRediT authorship contribution statement

**Peng Wu:** Writing – review & editing, Writing – original draft, Project administration, Funding acquisition. **Chengyun Zhang:** Writing – original draft, Data curation. **Yueyue Yin:** Writing – review & editing, Formal analysis. **Xiaobin Zhang:** Writing – review & editing. **Qi Li:** Writing – review & editing, Conceptualization. **Lijingyi Yuan:** Formal analysis, Data curation. **Yahe Sun:** Writing – review & editing, Resources, Project administration. **Shuhua Zhou:** Writing – review & editing, Resources, Project administration, Funding acquisition. **Jiayan Wu:** Writing – review & editing, Writing – original draft, Methodology, Data curation, Conceptualization.

## Declaration of competing interest

All authors disclosed no relevant relationships.
